# A National e-Health Program for the Prevention and Management of Overweight and Obesity in Childhood and Adolescence in Greece

**DOI:** 10.3390/nu12092858

**Published:** 2020-09-18

**Authors:** Athanasia Tragomalou, George Moschonis, Penio Kassari, Ifigeneia Papageorgiou, Sofia-Maria Genitsaridi, Sofia Karampatsou, Yannis Manios, Evangelia Charmandari

**Affiliations:** 1 Division of Endocrinology, Metabolism and Diabetes, First Department of Pediatrics, National and Kapodistrian University of Athens Medical School, “Aghia Sophia” Children’s Hospital, 11527 Athens, Greece; nansymou@hotmail.com (A.T.); peniokassari@gmail.com (P.K.); ifipap88@gmail.com (I.P.); sgenitsaridi@gmail.com (S.-M.G.); karampatsousi@gmail.com (S.K.); 2 Division of Endocrinology and Metabolism, Center of Clinical, Experimental Surgery and Translational Research, Biomedical Research Foundation of the Academy of Athens, 11527 Athens, Greece; 3 Department of Dietetics, Nutrition and Sport, School of Allied Health, Human Services and Sport, La Trobe University, Melbourne, VIC 3086, Australia; G.Moschonis@latrobe.edu.au; 4 Department of Nutrition and Dietetics, Harokopio University of Athens, Kallithea, 17671 Athens, Greece; manios@hua.gr

**Keywords:** overweight and obesity in childhood and adolescence, national e-health program, cardiometabolic risk factors

## Abstract

Obesity in childhood and adolescence represents one of the most challenging public health problems of the 21st century owing to its epidemic proportions worldwide and the associated significant morbidity, mortality and public health costs. In Greece, the prevalence of overweight and obesity in childhood and adolescence exceeds 30–35%. To address the increasing prevalence of overweight and obesity in children and adolescents in our country, we developed the ‘National e-Health Program for the Prevention and Management of Overweight and Obesity in Childhood and Adolescence’, which provides specific and detailed guidance to all primary health care physicians about the personalized management of children and adolescents with overweight or obesity. In the present study we evaluated 2400 children and adolescents [mean age ± SEM: 10.10 ± 0.09 years.; Males: 1088, Females: 1312; Obesity (*n* = 1370, 57.1%), Overweight (*n* = 674, 28.1%), normal BMI (*n* = 356, 14.8%)], who followed the personalized multi-disciplinary management plan specified by the ‘National e-Health Program for the Prevention and Management of Overweight and Obesity in Childhood and Adolescence’, and were studied prospectively for 1 year. We demonstrated that at the end of the first year, the prevalence of obesity decreased by 32.1%, the prevalence of overweight decreased by 26.7%, and the cardiometabolic risk factors improved significantly. These findings indicate that our National e-Health Program is effective at reducing the prevalence of overweight and obesity in childhood and adolescence after one year of intervention in the largest sample size reported to date.

## 1. Introduction

Obesity represents one of the most challenging public health problems of the 21st century owing to both its epidemic proportions worldwide and the associated significant morbidity and mortality [1]. During the last four decades, the prevalence of overweight and obesity in childhood and adolescence has risen substantially in most high-income countries and appears to be rising rapidly in low-income and middle-income countries [1,2,3]. According to the World Health Organization (WHO), 41 million children under the age of 5 years and more than 340 million children and adolescents aged 5–19 years are estimated to be overweight or obese [3]. In Greece, the prevalence of overweight and obesity in childhood and adolescence exceeds 30–35%, which is among the highest rates in Europe [4].

Although the adipose tissue was originally considered a storage organ for triacylglycerol, it has long been recognized as a metabolically active endocrine organ that affects various biological processes, such as energy homeostasis, nutrition, immunity, glucose concentrations, lipid metabolism and angiogenesis [5]. Obesity is characterized by a marked increase in the adipose tissue in the body and is defined by the Body Mass Index (BMI; the weight in kilograms divided by the square of the height in meters). Although it is not the most representative indicator of body composition, especially for muscular or very tall people, it is nevertheless the most widely used. According to the World Health Organization, for adults, overweight is defined as BMI between 25–30 kg/m^2^ and obesity as BMI > 30 kg/m^2^ [1,6]. In children and adolescents these cut-off limits are not clear and vary depending on chronological age and gender. Accordingly, in childhood and adolescence, overweight is defined as BMI between 85th–95th percentile, while obesity is defined as BMI > 95th percentile for age and gender [6].

Obesity is characterized by low-grade, systemic, chronic inflammation, and increased production and release of pro-inflammatory, atherogenic cytokines and oxidative stress [7]. Therefore, it is associated with several complications, including insulin resistance, dyslipidemia, hypertension, endothelial dysfunction, diabetes mellitus type 2, early onset atherosclerotic cardiovascular disease, hypogonadism, orthopedic problems, fatty liver disease, cholecystitis, social stigmatization, and increased incidence of malignancies [6,7,8,9,10,11]. Given that overweight and obesity in childhood and adolescence lead to obesity in adulthood, it is likely that the “obesity epidemic” in childhood may reverse the current decline in mortality owing to cardiovascular causes, and may lead to a shorter life span for today’s children [10]. In addition to the increased morbidity and mortality, overweight and obesity account for a significant increase in public health costs. The global economic impact from obesity is approximately $2.0 trillion USD or 2.8% of the global gross domestic product (GDP), which is almost equivalent to the global impact from smoking or armed violence, war and terrorism [12].

The progressively increasing prevalence of overweight and obesity in childhood and adolescence in Greece, and possibly other countries, indicates that our current health policies are not effective, and that further developments in health policy strategies are required. To address this problem in our country, we developed the ‘National e-Health Program for the Prevention and Management of Overweight and Obesity in Childhood and Adolescence’, which provides specific and detailed guidance to all primary health care physicians about the personalized management of overweight or obese children and adolescents [13,14]. The ‘National e-Health Program for the Prevention and Management of Overweight and Obesity in Childhood and Adolescence’ is a National Program available to all Pediatricians and General Practitioners in our country, who—through a web application—register online all children and adolescents nationwide, and receive guidance and detailed advice regarding their management depending on their BMI [13,15].

More specifically, using information and communication technologies, we developed an electronic medical records file (EMRF) for the electronic documentation of the present, past and family medical history, as well as the clinical examination findings of all children and adolescents. In addition, we developed the therapeutic algorithm files (TAF), which provide detailed information and guidance to Pediatricians and General Practitioners about the management of each child or adolescent. Upon entering the EMRF, each Pediatrician or General Practitioner has the opportunity to create a new EMRF in order to record a new patient, while he/she is able to view only the EMRFs of the patients under his/her care. In the EMRF, the physician records information on personal and demographic data, such as the social security number, name, surname, birth date and demographic data, the child’s present and past medical history, the family history, the clinical examination findings (including the anthropometric parameters and arterial blood pressure) and the patient’s signed consent form [13,15]. The electronic database system (EDS) then automatically calculates the BMI from the data on height and weight and informs the physician whether the patient has normal or increased BMI. In addition, the corresponding growth chart for BMI appears on the computer screen. Subsequently, the EDS selects the most appropriate therapeutic algorithm file (TAF), according to the patient’s age, gender, BMI and all other information (including information on diet and exercise) entered in the system. Therefore, the TAF provides a comprehensive and personalized multidisciplinary management plan for the prevention and/or management of overweight and obesity for the patient. The TAF indicates what the initial advice to the patient and his/her family should be; when the physician should reassess the patient; how he/she will manage the patient if there is adequate response to the therapeutic interventions or if there is no response to the therapeutic interventions despite compliance with those; when he/she will perform laboratory investigations and which ones; when he/she will refer the patient to a pediatric dietician or psychologist; and when he/she will refer the patient to a pediatric endocrinologist or a specialist center with expertise in the management of overweight and obesity [13,15].

To the best of our knowledge, our ‘National e-Health Program for the Prevention and Management of Overweight and Obesity in Childhood and Adolescence’ is the first web-based e-Health application in Greece developed to be used by health professionals. It is accessible by the following URL: https://app.childhood-obesity.gr/ and has been awarded the BRONZE Award in the category “e-Health/H1.1 Digital Applications for Integrated Patient care” by the Healthcare Business Awards in 2016.

The aim of the present study was to evaluate the effectiveness of the interventions suggested by this electronic system in reducing the prevalence of obesity and overweight, and to present the progress of a large number of children and adolescents who have followed the personalized multi-disciplinary management plan specified by the ‘National e-Health Program for the Prevention and Management of Overweight and Obesity in Childhood and Adolescence’.

## 2. Patients and Methods

### 2.1. Patients

Two thousand four hundred (*n* = 2400) children and adolescents, aged 2–18 years, (mean age ± SEM: 10.10 ± 0.09 years; 1088 males, 1312 females) attending our Out-patient Clinic for the Prevention and Management of Overweight and Obesity in Childhood and Adolescence were studied prospectively for one year. Subjects were classified as having obesity, overweight or normal BMI according to the International Obesity Task Force (IOTF) cut-off points [16]. The clinical characteristics of all subjects are summarized in Table 1. The study was approved by the local Committee on the Ethics of Human Research. (Approval Number: EB-PASCH-MoM: 28/11/2013, Re: 10290-14/05/2013). Written informed consent was obtained in all cases by a parent/guardian, and assent was given by children older than 7 years.

### 2.2. Methods

All participants were admitted to the Endocrine Unit early in the morning on the day of the study, and a detailed medical history and clinical examination, including pubertal assessment and standard anthropometric measurements (weight, height, waist circumference, hip circumference) were obtained by a single trained observer. Body weight was measured in light clothing and without shoes using the same scale for all subjects (Seca GmbH & Co. KG., Hamburg, Germany). Standing height was also measured without shoes using a stadiometer (Holtain Limited, Crymych-Dyfed, UK). Waist and hip circumferences were measured according to WHO STEPS protocol using the same stretch-resistant tape (Seca GmbH & Co. KG., Hamburg, Germany) with the subject on standing position. More specifically, waist circumference was measured in the horizontal plane midway between the lowest rib and the iliac crest at the end of a normal expiration. Hip circumference was measured in the horizontal plane at the level of maximum circumference of hips and buttocks. On both occasions, the tape did not compress the skin and was parallel to the floor. Blood pressure was determined by a sphygmomanometer (Comfort 20/40, Visomat, Parapharm, Metamorphosi, Attiki, Greece) and with an appropriate cuff according to the age of the subject [14].

A blood sample for baseline hematological, biochemical and endocrinologic investigations was drawn at 8:00 h following a 12-h fast. Samples were centrifuged and separated immediately after collection and were stored at −80 °C until assayed.

### 2.3. Assessment and Interventions

At initial assessment, all subjects were evaluated by a Pediatrician and Pediatric Dietitian for their daily eating habits, and a 24-h recall of their diet was performed based on the USDA method [17]. The dietitian recorded the number of meals and snacks, the usual food choices, the person responsible for the preparation of meals, the amount of liquids (water, milk, juices and other beverages) consumed, as well as the frequency and amount of junk food and sweet consumption. Subsequently, children and their parents were informed about the complications of obesity and the need for the whole family to adopt a healthier lifestyle. Also, they were guided about changes in their nutritional habits. They were given advice on a healthy diet according to “My Plate” standard, a visualized approach of the USDA 2010 guidelines [18], which included three main meals (breakfast, lunch and dinner) and two snacks (fruits, vegetables) at mid-morning and mid-afternoon. The importance of breakfast consumption was emphasized because of its association with better cognitive performance at school, as well as with better achievement and maintenance of normal weight. The appropriate food portions were determined according to the guidelines proposed by the National Nutrition Guide for Infants, Children and Adolescents [19]. The aim was to recommend a personalized plan of healthy diet, which would also take into consideration the child’s preferences on food consumption (and therefore not be perceived as boring or difficult), as well as the food availability and preparation while the child is at school or at home.

In addition, a professional fitness Personal Trainer evaluated children and adolescents in relation to their activities and hobbies throughout the week, suggested a personalized exercise program, and encouraged the whole family to avoid a sedentary lifestyle and to follow a physical activity of their choice on a daily basis for 30–45 min, such as walking, jogging, dancing, cycling [20]. The Personal Trainer discussed the child’s interests with the family in order to identify suitable sport activities. The aim was to recommend a personalized physical activity plan, which would not be perceived as compulsory, boring or difficult, but rather as a highly enjoyable and entertaining activity. The assessment of the Personal Trainer was repeated each month and recorded all information about physical activity and exercise.

Finally, subjects referred for psychological evaluation were assessed by a Pediatric Clinical Psychologist, who evaluated the family dynamics and provided psychological support to children and their parents. In cases where more severe psychopathology was evident, patients were referred to a mental health service.

All subjects included in the study complied with the advice given on diet and exercise, as reported by them and their families. Patients who required psychological or psychiatric input were excluded from the study. Subjects with obesity were followed-up at least every month, with overweight every two months and with normal-BMI every three months. It should be emphasized that all information and guidance on life-style interventions were given in person, when our patients attended our Out-patient Clinic, and were the same with those provided by the National e-Health Program for the Prevention and Management of Overweight and Obesity in Childhood and Adolescence. At each subsequent appointment, the Pediatrician re-evaluated the anthropometric measurements and the goals set in previous sessions were discussed in detail with the Pediatric Dietitian and the Personal Trainer, as well as the possible difficulties faced by children in achieving their optimal BMI. Detailed hematologic, biochemical and endocrinologic investigations were performed at the beginning and at the end of the study.

### 2.4. Assays

Standard hematologic investigations were determined using the ADVIA 2110i analyzer (Roche Diagnostics GmbH, Mannheim, Germany). The concentrations of glucose, total cholesterol, triglycerides and high-density lipoprotein cholesterol (HDL) were determined using the ADVIA 1800 Siemens analyzer (Siemens Healthcare Diagnostics, Tarrytown, NY, USA). Apolipoprotein A1, B (ApoA1, ApoB) and lipoprotein (a) concentrations were determined by means of latex particle-enhanced immunonephelometric assays on the BN ProSpec nephelometer (Dade Behring, Siemens Healthcare Diagnostics, Liederbach, Germany). Insulin was measured using automated electro-chemiluminescense immunoassays (Analyzer Cobas e411, Roche Diagnostics GmbH). Insulin-like growth factor-I and insulin-like growth factor binding protein-3 were measured using automated chemiluminescence immunoassays on an IMMULITE 2000 immunoassay system (Siemens Healthcare Diagnostics Products Ltd., Frimley, Camberley, UK). Total 25-hydroxyvitamin D was measured using an automated electrochemiluminescence immunoassay on the Modular Analytics E170 analyzer. Hemoglobin A1C (HbA1C) was determined using reversed-phase cation exchange high-performance liquid chromatography on an automated glycohemoglobin analyzer HA-8160 (Arkray, Kyoto, Japan).

### 2.5. Statistical Analyses

The results are presented as mean value ± standard error of the mean (SEM) for continuous variables and frequencies (%) for categorical variables. Normality was tested by using graphical methods (i.e., histograms and Q–Q plots) and homogeneity of variance was tested with the Levene’s test. Differences in the distribution of continuous variables between the three categories of BMI (obesity, overweight and normal BMI) were assessed using the ANOVA F test. The Bonferroni rule was applied to each of the multiple comparisons for adjustment for a significance level of 5%. The comparison of pre- and post-intervention results within each category of BMI was calculated by a paired samples *t* test for normally distributed variables. The associations between skewed variables and groups of participants were evaluated by the Mann-Whitney U test, the Kruskal-Wallis H test or Wilcoxon’s signed rank test. The associations between categorical variables were analyzed using Pearson’s χ2 or the Monte Carlo test. A series of two-sample z tests of proportions was conducted to determine whether there were significant differences between groups. Data were analyzed using the SPSS statistical package version 24.0 (SPSS Inc., Chicago, IL, USA).

## 3. Results

The study sample consisted of 2400 children and adolescents (mean age ± SEM: 10.10 ± 0.09 years.; 1088 males, 1312 females), who were studied prospectively for 1 year. Subjects were classified as having obesity (*n* = 1370, 57.1%), overweight (*n* = 674, 28.1%) or normal BMI (*n* = 356, 14.8%) according to the International Obesity Task Force cut-off points [15]. The clinical characteristics of all subjects at baseline are shown in Table 1.

**Figure 1:**
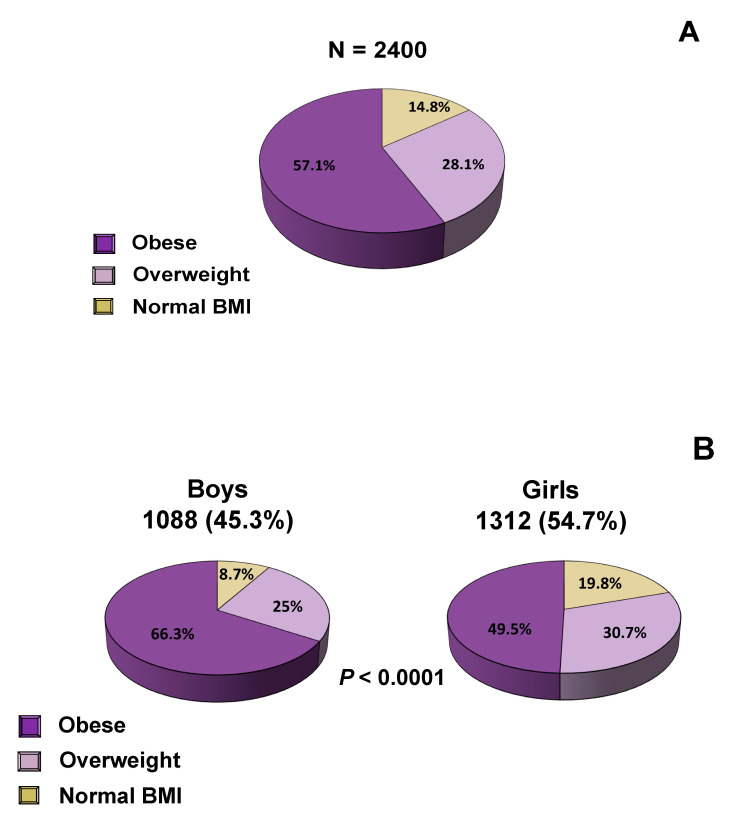
Distribution of BMI: (**A**) in all subjects, (**B**) according to gender.

At initial evaluation, the percentage of subjects with obesity was 57.1%, overweight 28.1% and normal-BMI 14.8% (Figure 1A). A significantly higher number of boys had obesity compared with girls (66.3% vs. 49.5%, *p* < 0.0001), while a higher number of girls had overweight compared with boys (30.7% vs. 25%, *p* < 0.0001) (Figure 1B). There was no significant difference in BMI category between prepubertal and pubertal children. The clinical characteristics of all subjects at baseline are shown in Table 1 and after one year of intervention in Table 2. Children and adolescents with obesity had significantly higher systolic (SBP) and diastolic (DBP) blood pressure, waist circumference, hip circumference, waist-to-height ratio and waist-to-hip ratio than their overweight and normal-BMI counterparts. In addition, a strong positive correlation was observed between children’s BMI and their parents’ BMI, indicating that parents with overweight or obesity were more likely to have children with overweight or obesity (Table 1 and Table 2). Table 3 presents the biochemical and endocrinologic parameters. Subjects with obesity had significantly higher concentrations of fasting plasma glucose and serum insulin, HbA1C, triglycerides, LDL-cholesterol, uric acid and ApoB, and significantly lower concentrations of HDL-cholesterol and ApoA1 than their overweight and normal-BMI counterparts (Table 3). Table 4 presents only the subjects who had a lipid profile indicative of dyslipidemia at baseline, while Table 8 presents the improvement that was demonstrated in terms of dyslipidemia one year after implementation of our interventions. The subgroup of subjects who had dyslipidemia was determined according to the American College of Cardiology criteria for dyslipidemia [21].

**Figure 2:**
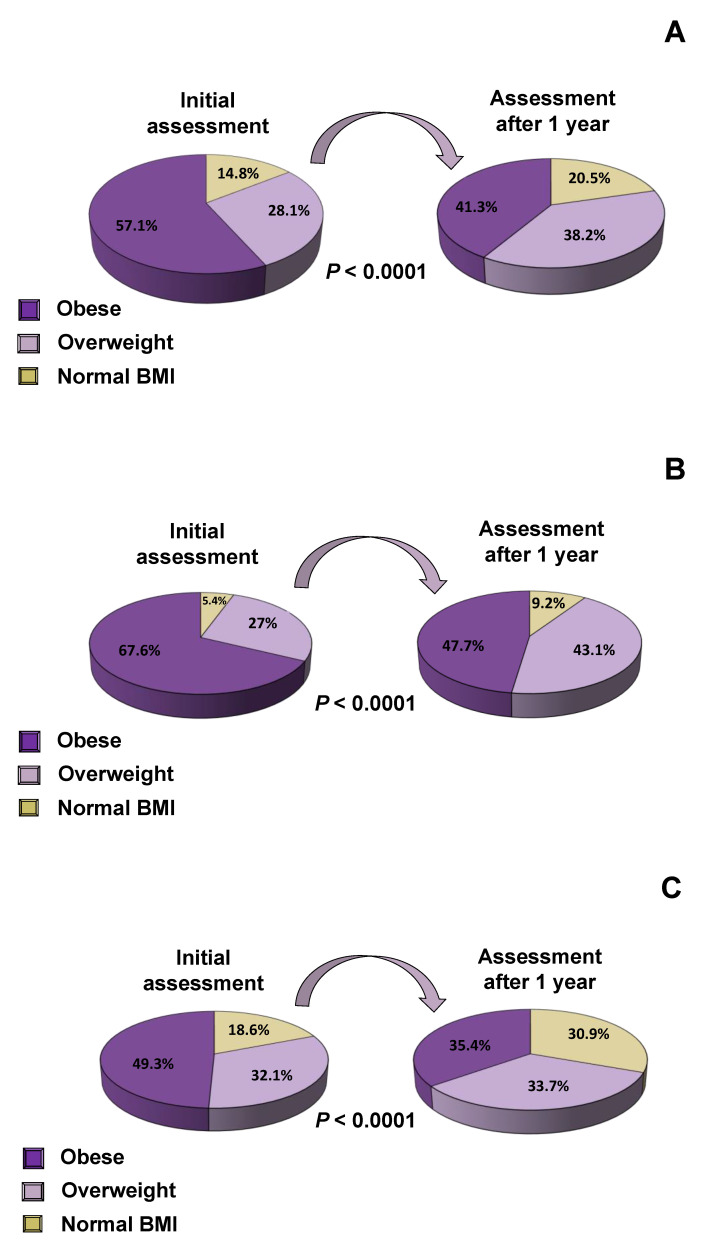
Alteration in BMI after 1 year of intervention: (**A**) in all subjects, (**B**) in boys, (**C**) in girls.

**Figure 3:**
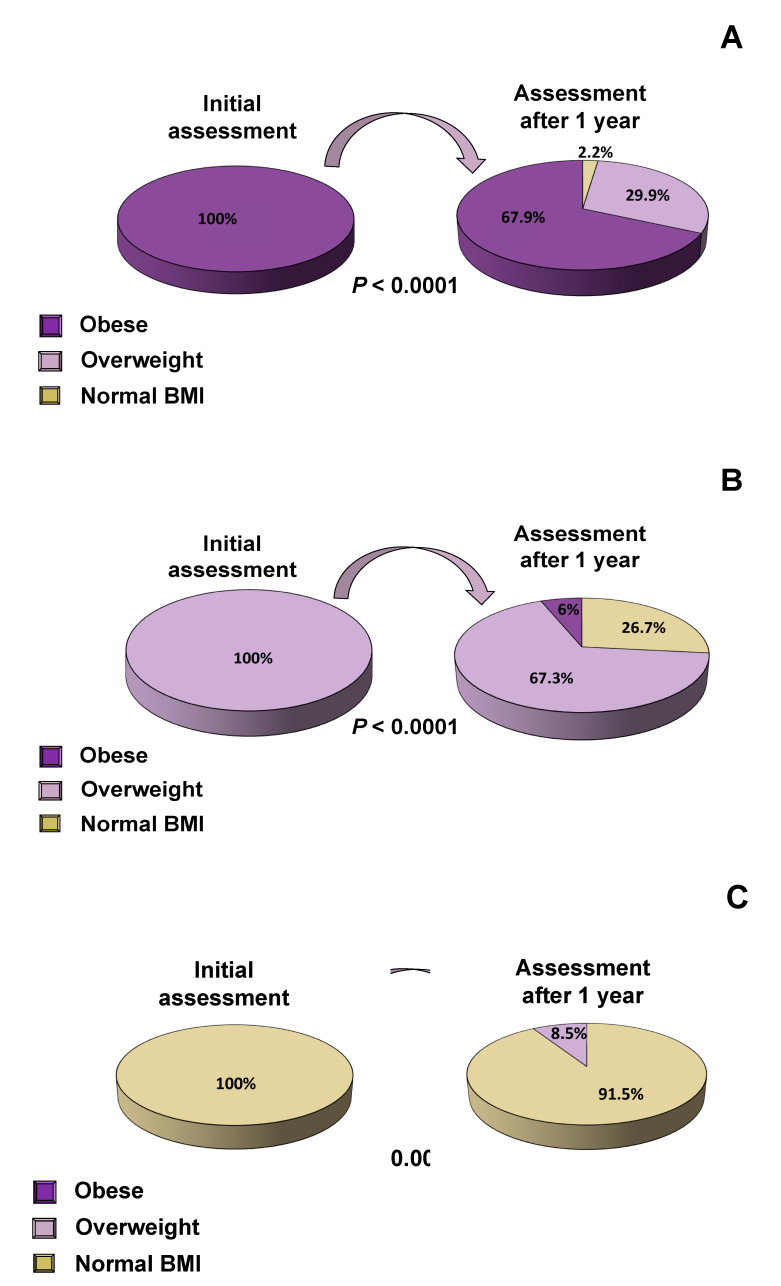
Alteration of BMI after 1 year of intervention: (**A**) in obese children and adolescents, (**B**) in overweight children and adolescents, (**C**) in normal BMI children and adolescents.

Following one year of intervention, the proportion of obese subjects decreased significantly by 15.8% (57.1% vs. 41,3%, *p* < 0.0001), while the proportion of overweight and normal-BMI subjects increased significantly by 10.1% and 5.7%, respectively (28.1% vs. 38.2%, 14.8% vs. 20.5%, *p* < 0.0001 all) (Figure 2A). Similar changes were observed in both boys and girls (Figure 2B,C, respectively). Parameters that are associated with cardiovascular risk factors, such as lipid profile, plasma glucose, serum insulin and HbA1C concentrations were evaluated at baseline and one year after the interventions in subjects with obesity (Table 5), overweight (Table 6) and normal BMI (Table 7). When the progress of subjects was evaluated after one year of implementation of the multi-disciplinary management interventions, the proportion of subjects with obesity decreased by 32.1% while the proportion of subjects with overweight decreased by 26.7% (Figure 3A,B). The majority of children and adolescents with normal BMI maintained their normal BMI till the end of our study, and only a small percentage of 8.5% developed overweight (Figure 3C). The cardiometabolic risk indices improved significantly, as indicated by the improvement in the lipid profile and the criteria for dyslipidemia (Table 8). More specifically, we divided our population of children and adolescents into different subgroups, who fulfilled the criteria for dyslipidemia according to American College of Cardiology guidelines [21], and studied them at the beginning and the end of the study. We demonstrated that one year after implementation of our interventions, total cholesterol, LDL, triglycerides and ApoB decreased significantly, while HDL increased significantly, most likely as a result of the reduction in BMI (Table 8).

## 4. Discussion

In the present study we evaluated 2400 children and adolescents attending our Out-patient Clinic for the Prevention and Management of Overweight and Obesity. The majority of these subjects had obesity (57.1%) or overweight (28.1%), while only a small proportion had normal BMI (14.8%). All subjects followed the personalized multi-disciplinary management plan specified by the ‘National e-Health Program for the Prevention and Management of Overweight and Obesity in Childhood and Adolescence’ and were assessed at the end of the first year of their participation in this program. We demonstrated that at the end of the first year the proportion of subjects with obesity decreased by 32.1%, the proportion of subjects with overweight decreased by 26.7%, and the cardiometabolic risk factors improved significantly. These findings indicate that our National e-Health Program is effective at reducing the prevalence of overweight and obesity in childhood and adolescence after one year of intervention in the largest sample size reported to date.

In addition to the observed improvement in BMI, a significant improvement was also observed in cardiovascular risk factors, such as lipid profile, plasma glucose, serum insulin and HbA1C concentrations. In particular, the concentrations of HDL-cholesterol and the anti-inflammatory cytokine, adiponectin, increased significantly, while the concentrations of LDL-cholesterol, Apo-B and HbA1C decreased significantly as a result of the reduction in BMI. These encouraging results demonstrate the importance of such a coordinated effort within the context of a multi-disciplinary intervention program in the effective management of overweight and obesity in childhood and adolescence, as well as the prevention of atherosclerotic cardiovascular disease later in life. The increased adipose tissue and its associated dyslipidemia and hypertension are the underlying pathogenetic factors in the development of endothelial dysfunction, increased fat deposition in the aortic linear muscle, increased arterial wall thickness and abnormalities in the coronary arteries, already present in childhood and adolescence [22,23]. Indeed, less than 1% of adults who did not have risk factors in childhood eventually developed carotid plaque in adulthood [24]. In addition, the cardiovascular risk is directly related to circulating atherogenic factors, i.e., lipoproteins that interact and invade the vessel wall, and is determined by the balance between proathrogens (ApoB) and antiplatelet-antiatherogenic agents (ApoA1) [25]. Therefore, this study allowed us to document the cardiovascular risk factors associated with dyslipidemia in a large sample of children and adolescents, as well as the significant improvement that was observed as a result of complying with the interventions specified by our National e-Health Program.

Furthermore, we confirmed the expected higher SBP and DBP of children and adolescents with obesity compared to their overweight and normal-BMI counterparts. Obesity is one of the most important factors in the development of primary (idiopathic) hypertension in childhood. Hypertension, in turn, is associated with left ventricular hypertrophy and early cardiovascular disease [26,27]. Therefore, adequately addressing the epidemic of obesity in childhood and adolescence is of major importance in reducing systemic inflammation [28,29] and atherosclerotic cardiovascular disease in adulthood. Maintaining normal BMI and adopting a healthy lifestyle have been the main goals of our program that seem to have been largely achieved.

Addressing the obesity epidemic should include measures that take into consideration genetic, epigenetic and environmental factors [30]. In addition to genetic factors, the life-style of the parents influences the weight of their children. Longitudinal studies showed that parental obesity is strongly associated with childhood obesity, and this effect is more pronounced in adolescence [31,32]. Furthermore, consumption of a high-fat diet by the parents even prior to conception may result in increased weight gain of the child [23,33]. Therefore, it is important to determine the various genetic, epigenetic or environmental factors that affect weight gain [31,34,35,36].

In summary, the “National e-Health Program for the Prevention and Management of Overweight and Obesity in Childhood and Adolescence”, is a unique and innovative e-Health application that provides a comprehensive, personalized, multidisciplinary intervention program to combat overweight and obesity in childhood and adolescence in Greece. Evaluation of this program in 2400 children and adolescents indicated that it is effective at reducing the prevalence of overweight and obesity in childhood and adolescence, and at improving the cardiometabolic risk factors after one year of intervention.

## Figures and Tables

**Table 1:** Clinical characteristics of all subjects at initial assessment.

	Obesity	Overweight	Normal-BMI	*p*-Value
**Age (years)**	10.10 ± 0.09	10.20 ± 0.11	9.80 ± 0.16	0.147
**Weight (Kg)**	62.78 ± 0.65	49.41 ± 0.54	38.79 ± 0.73	**<0.0001**
**Height (cm)**	144.47 ± 0.52	143.43 ± 0.58	139.03 ± 0.94	**<0.0001**
**BMI (kg/m^2^)**	28.70 ± 0.14	23.40 ± 0.09	19.31 ± 0.16	**<0.0001**
**Waist/Hip ratio**	0.95 ± 0.01	0.93 ± 0.01	0.88 ± 0.01	**<0.0001**
**Waist/Height**	0.61 ± 0.003	0.55 ± 0.003	0.51 ± 0.005	**<0.0001**
**SBP**	114 ± 0.39	110 ± 0.45	105 ± 0.62	**<0.0001**
**DBP**	67 ± 0.32	66 ± 0.75	63 ± 0.50	**<0.0001**
	*n* (%)	*n* (%)	*n* (%)	
**Pubertal status**				
Prepubertal	732 (57.1)	367 (28.6)	183 (14.3)	0.829
Pubertal	599 (57)	294 (27.9)	159 (15.1)	
**Mother’s BMI**				
Normal BMI	300 (41.7)	249 (34.6)	170 (23.6)	**<0.0001**
Overweight	331 (61)	142 (26.2)	70 (12.9)	
Obesity	367 (69.4)	109 (20.6)	53 (10)	
**Father’s BMI**				
Normal BMI	147 (41.2)	124 (34.7)	86 (24.1)	**<0.0001**
Overweight	388 (53.2)	228 (31.3)	113 (15.5)	
Obesity	455 (65.8)	149 (21.5)	88 (12.7)	

Abbreviations: DBP: Diastolic Blood Pressure, SBP: Systolic Blood Pressure; Continuous variables are presented as mean ± standard error of the mean (SEM) and categorical as frequencies (percentages); *p* values were derived by comparisons between the three categories of BMI using one-way ANOVA; Pearson’s χ^2^ for categorical variables; Statistically significant associations are shown in bold.

**Table 2:** Clinical characteristics of all subjects after a year of intervention.

	Obesity	Overweight	Normal-BMI	*p*-Value
**Age (years)**	11.20 ± 0.16	10.96 ± 0.14	11.18 ± 0.20	0.345
**Weight (Kg)**	68 ± 1.15	53.60 ± 0.77	45 ± 0.85	**<0.0001**
**Height (cm)**	150.47 ± 0.87	149.11 ± 0.80	147 ± 1.04	**0.008**
**BMI (kg/m^2^)**	28.87 ± 0.24	23.53 ± 0.12	20.37 ± 0.17	**<0.0001**
**Waist/Hip ratio**	0.93 ± 0.005	0.91 ± 0.005	0.86 ± 0.007	**<0.0001**
**Waist/Height**	0.60 ± 0.004	0.53 ± 0.003	0.49 ± 0.005	**<0.0001**
**SBP**	115 ± 0.79	111 ± 0.66	107 ± 0.85	**<0.0001**
**DBP**	69 ± 0.62	67 ± 0.59	65 ± 0.69	**<0.0001**
	*n* (%)	*n* (%)	*n* (%)	
**Pubertal status**				
Prepubertal	107 (42.3)	103 (40.7)	43 (17)	**0.043**
Pubertal	165 (38.6)	155 (36.2)	108 (25.2)	
**Mother’s BMI**				
Normal BMI	88 (29.6)	118 (39.7)	91 (30.6)	**<0.0001**
Overweight	95 (42.2)	84 (37.3)	46 (20.4)	
Obesity	136 (53.8)	89 (35.2)	28 (11.1)	
**Father’s BMI**				
Normal BMI	41 (27.3)	59 (39.3)	50 (33.3)	**<0.0001**
Overweight	117 (37)	126 (39.9)	73 (23.1)	
Obesity	157 (52.3)	104 (34.7)	39 (13)	

Abbreviations: DBP: Diastolic Blood Pressure, SBP: Systolic Blood Pressure WHratio: Waist to Hip ratio, WHtR: Waist to Height ratio; Continuous variables are presented as mean ± standard error of the mean (SEM) and categorical as frequencies (percentages); *p* values were derived by comparisons between the three categories of BMI using one-way ANOVA; Pearson’s χ^2^ for categorical variables; Statistically significant associations are shown in bold.

**Table 3:** Biochemical and endocrinologic parameters in all subjects at baseline.

	Obesity	Overweight	Normal-BMI	*p*-Value
**Glucose (mg/dL)**	80.20 ± 0.28	78.72 ± 0.34	79.11 ± 0.43	**0.003**
**Cholesterol (mg/dL)**	157.11 ± 0.81	158.45 ± 1.17	159.71 ± 1.53	0.290
**TG (mg/dL)**	82.38 ± 1.28	73.99 ± 1.77	64.35 ± 1.79	**<0.0001**
**HDL (mg/dL)**	50.05 ± 0.36	53.67 ± 0.53	59.51 ± 0.87	**<0.0001**
**LDL (mg/dL)**	91.76 ± 0.72	90.96 ± 1.04	87.60 ± 1.32	**0.015**
**ApoA1 (mg/dL)**	138.86 ± 0.62	142.86 ± 0.89	149.76 ± 1.33	**<0.0001**
**ApoB (mg/dL)**	75.93 ± 0.53	73.69 ± 0.77	71.52 ± 0.88	**<0.0001**
**Lp(a) (mg/dL)**	17.46 ± 0.68	17.02 ± 0.97	17.98 ± 1.42	0.874
**IGF-I (ng/mL)**	301.87 ± 5.10	306.64 ± 7.1	305.73 ± 10.66	0.517
**IGFBP-3 (μg/mL)**	5.09 ± 0.03	5.01 ± 0.04	4.82 ± 0.07	**0.001**
**Insulin (μUI/mL)**	17.39 ± 0.32	12.50 ± 0.29	9.45 ± 0.34	**<0.0001**
**HbA1C (%)**	5.26 ± 0.01	5.21 ± 0.01	5.18 ± 0.01	**<0.0001**

Abbreviations: ApoA1: Apolipoprotein A1, ApoB: Apolipoprotein B, HbA1c: Haemoglobin A1c, HDL: High-Density Lipoprotein, IGF-I: Insulin-like Growth Factor 1, IGF-BP3: IGF-binding protein 3, LDL: Low-Density Lipoprotein, Lp(a): Lipoprotein a, TG: Triglycerides, Continuous variables are presented as mean ± standard error of the mean (SEM); *p* values were derived by comparisons between the three categories of BMI using one-way ANOVA for normally distributed variables and Kruskal-Wallis H test for skewed variables; Statistically significant associations are shown in bold.

**Table 4:** Lipid profile indicative of dyslipidemia at baseline.

	Obesity	Overweight	Normal-BMI	*p*-Value
**Cholesterol (≥200 mg/dL)**	213.98 ± 1.72	218.54 ± 3.15	218.63 ± 3.76	0.297
**LDL (≥130 mg/dL)**	146.66 ± 2.34	146.47 ± 3.45	141.31 ± 4.26	0.598
**ApoB (≥110 mg/dL)**	124.69 ± 2.08	129.94 ± 4.87	117 ± 5.13	0.400
**Triglycerides**				
**0–9 age (≥100 mg/dL)**	133.95 ± 4.30	146.62 ± 8.87	123.29 ± 8.11	0.496
**10–19age (≥30 mg/dL)**	176.60 ± 5.73	180.55 ± 12.96	159.67 ± 14.82	0.152
**HDL (<40 mg/dL)**	35.04 ± 0.25	35.53 ± 0.43	34.23 ± 0.96	0.340
**ApoA1 (<115 mg/dL)**	104.93 ± 0.88	107.49 ± 0.98	105.07 ± 2.24	0.241
**Lp(a) (>30 mg/dL)**	57.23 ± 1.71	61.21 ± 2.74	61.97 ± 3.77	0.327

Abbreviations: ApoA1: Apolipoprotein A1, ApoB: Apolipoprotein B, HDL: High-Density Lipoprotein, LDL: Low-Density Lipoprotein, Lp(a): Lipoprotein a.

**Table 5:** Biochemical and endocrinologic parameters in subjects with obesity.

	Initial Assessment (Mean ± SEM)	Annual Assessment (Mean ± SEM)	*p*-Value
**Glucose (mg/dL)**	80.11 ± 0.36	80.54 ± 0.10	0.269
**Cholesterol (mg/dL)**	158.57 ± 1.21	157.36 ± 1.38	0.056
**Triglycerides (mg/dL)**	82.36 ± 1.81	81.07 ± 1.84	0.657
**HDL (mg/dL)**	50.23 ± 0.53	53.28 ± 0.60	**<0.0001**
**LDL (mg/dL)**	92.39 ± 1.05	89.37 ± 1.14	**<0.0001**
**ApoA1 (mg/dL)**	140.55 ± 0.96	141.60 ± 1.03	0.163
**ApoB (mg/dL)**	75.59 ± 0.91	73.48 ± 0.78	**<0.0001**
**Lp(a) (mg/dL)**	16.45 ± 0.99	15.29 ± 1.05	**0.031**
**Adiponectin (ng/mL)**	20,705.41 ± 1203.62	23,115.45 ± 1239.64	**0.008**
**Insulin (μUI/mL)**	17.52 ± 0.50	16.53 ± 0.42	0.321
**HbA1C (%)**	5.28 ± 0.01	5.24 ± 0.01	**<0.0001**

Abbreviations: ApoA1: Apolipoprotein A1, ApoB: Apolipoprotein B, HbA1C: Hemoglobin A1C, HDL: High-Density Lipoprotein, LDL: Low-Density Lipoprotein, Lp(a): Lipoprotein a.

**Table 6:** Biochemical and endocrinologic parameters in subjects with overweight.

	Initial Assessment (Mean ± SEM)	Annual Assessment (Mean ± SEM)	*p*-Value
**Glucose (mg/dL)**	78.05 ± 0.51	80.55 ± 0.50	**<0.0001**
**Cholesterol (mg/dL)**	157.96 ± 1.76	156.91 ± 1.87	0.442
**Triglycerides (mg/dL)**	73.49 ± 2.64	74.63 ± 2.68	0.258
**HDL (mg/dL)**	54.94 ± 3.16	55.79 ± 0.85	**<0.0001**
**LDL (mg/dL)**	90.52 ± 1.55	87.50 ± 1.87	**<0.0001**
**ApoA1 (mg/dL)**	140.72 ± 1.40	143.15 ± 1.35	0.087
**ApoB (mg/dL)**	73.61 ± 1.23	71.19 ± 1.11	**0.042**
**Lp(a) (mg/dL)**	14.55 ± 1.26	14.18 ± 1.38	**0.026**
**Adiponectin (ng/mL)**	24,436.87 ± 1865.23	29,474.32 ± 2110.22	**0.004**
**Insulin (μUI/mL)**	12.08 ± 0.40	13.14 ± 0.46	**0.006**
**HbA1C (%)**	5.23 ± 0.01	5.21 ± 0.02	**0.018**

Abbreviations: ApoA1: Apolipoprotein A1, ApoB: Apolipoprotein B, HbA1C: Haemoglobin A1C, HDL: High-Density Lipoprotein, LDL: Low-Density Lipoprotein, Lp(a): Lipoprotein a.

**Table 7:** Biochemical and endocrinologic parameters in subjects with normal-BMI.

	Initial Assessment (Mean ± SEM)	Annual Assessment (Mean ± SEM)	*p*-Value
**Glucose (mg/dL)**	78.15 ± 0.73	79.06 ± 0.63	0.052
**Cholesterol (mg/dL)**	161.45 ± 2.66	158.11 ± 2.66	**0.020**
**Triglycerides (mg/dL)**	67.23 ± 3.15	69.27 ± 3.88	0.918
**HDL (mg/dL)**	57.89 ± 1.32	60.68 ± 1.49	**0.002**
**LDL (mg/dL)**	90.04 ± 2.26	83.76 ± 2.22	**<0.0001**
**ApoA1 (mg/dL)**	148.97 ± 2.02	147.79 ± 2.12	0.575
**ApoB (mg/dL)**	71.13 ± 1.42	69.43 ± 1.50	0.167
**Lp(a) (mg/dL)**	14.71 ± 1.89	15.90 ± 2.10	**0.042**
**Adiponectin (ng/mL)**	18,555.65 ± 2867.98	27,270.78 ± 3133.59	**0.002**
**Insulin (μUI/mL)**	12.08 ± 0.40	13.14 ± 0.46	**0.006**
**HbA1C (%)**	5.23 ± 0.01	5.21 ± 0.02	**0.018**

Abbreviations: ApoA1: Apolipoprotein A1, ApoB: Apolipoprotein B, HbA1C: Haemoglobin A1C, HDL: High-Density Lipoprotein, LDL: Low-Density Lipoprotein, Lp(a): Lipoprotein a.

**Table 8:** Lipid profile alterations indicative of dyslipidemia following the multidisciplinary interventions.

	Initial Assessment	Annual Assessment	*p*-Value
**Cholesterol (≥200mg/dL)**	221.23 ± 3.01	202.06 ± 4.22	**<0.0001**
**LDL (≥130mg/dL)**	142.33 ± 2	122.17 ± 3.02	**<0.0001**
**ApoB (≥110 mg/dL)**	130.16 ± 3.17	97.87 ± 4.61	**<0.0001**
**Triglycerides**			
**0–9 age (≥100 mg/dL)**	130.60 ± 4.83	100.79 ± 5.70	**<0.0001**
**10–19 age (≥130 mg/dL)**	174.68 ± 5.68	125.34 ± 7.77	**<0.0001**
**HDL (<40 mg/dL)**	35.39 ± 0.30	41.61 ± 0.74	**<0.0001**
**ApoA1 (<115 mg/dL)**	95.36 ± 7.70	99.36 ± 1.59	0.629
**Lp(a) (>30 mg/dL)**	58.77 ± 2.09	57.68 ± 2.90	0.113

Abbreviations: ApoA1: Apolipoprotein A1, ApoB: Apolipoprotein B, HDL: High-Density Lipoprotein, LDL: Low-Density Lipoprotein, Lp(a): Lipoprotein a.
